# Chikungunya Virus as Cause of Febrile Illness Outbreak, Chiapas, Mexico, 2014

**DOI:** 10.3201/eid2111.150546

**Published:** 2015-11

**Authors:** Tiffany F. Kautz, Esteban E. Díaz-González, Jesse H. Erasmus, Iliana R. Malo-García, Rose M. Langsjoen, Edward I. Patterson, Dawn I. Auguste, Naomi L. Forrester, Rosa Maria Sanchez-Casas, Mauricio Hernández-Ávila, Celia M. Alpuche-Aranda, Scott C. Weaver, Ildefonso Fernández-Salas

**Affiliations:** University of Texas Medical Branch, Galveston, Texas, USA (T.F. Kautz, J.H. Erasmus, R.M. Langsjoen, E.I. Patterson, D.I. Auguste, N.L. Forrester, S.C. Weaver);; Universidad Autónoma de Nuevo León, San Nicolás de los Garza, Mexico (E.E. Diaz-Gonzalez);; Centro Regional de Investigación en Salud Pública, Tapachula, Mexico (I.R. Malo-García, I. Fernandez-Salas);; Universidad Autónoma de Nuevo León, Escobedo, Mexico (R.M. Sanchez-Casas);; Instituto Nacional de Salud Pública, Cuernavaca, México (M. Hernández-Ávila, C.M. Alpuche-Aranda)

**Keywords:** alphavirus, Aedes, arboviruses, arthralgia, chikungunya virus, disease outbreaks, Mexico, Pan American Health Organization, phylogeny, public health, viruses, vector-borne infections

## Abstract

Since chikungunya virus (CHIKV) was introduced into the Americas in 2013, its geographic distribution has rapidly expanded. Of 119 serum samples collected in 2014 from febrile patients in southern Mexico, 79% were positive for CHIKV or IgM against CHIKV. Sequencing results confirmed CHIKV strains closely related to Caribbean isolates.

Chikungunya virus (CHIKV), an arbovirus in genus *Alphavirus*, family *Togaviridae*, has undergone a rapid geographic expansion during the past decade (*1*). CHIKV is the causative agent of chikungunya fever, which may be accompanied by severe, debilitating, and even chronic arthralgia. During urban outbreaks, CHIKV uses the highly susceptible and anthropophilic *Aedes aegypti* and *A. albopictus* mosquitoes as vectors, which results in high attack rates (*1*).

The West African and East/Central/South African (ECSA) CHIKV lineages, the 2 most ancient enzootic lineages, primarily circulate in sub-Saharan Africa. Over the past century, the ECSA lineage has given rise to the Asian lineage, found in urban cycles in India and Southeast Asia, and later to the Indian Ocean lineage, which emerged from Kenya into the Indian Ocean Basin in 2004 (*1*). In late 2013, an outbreak of chikungunya fever caused by an Asian lineage strain began in the Caribbean island of St. Martin (*2*,*3*). During 2014, CHIKV spread throughout the Caribbean and into Latin America, causing epidemics in South and Central America, while also causing sporadic autochthonous cases in North America (*1*). The importation from Angola and local circulation of an ECSA strain was also recently reported in Brazil (*4*), which now has 2 CHIKV lineages circulating. The total number of suspected cases in the Americas now exceeds 1.6 million and is steadily rising (*3*,*5*).

In October 2014, physicians in Chiapas State, Mexico, noticed large numbers of patients reporting febrile illness accompanied by rash and an unusual arthralgia, and chikungunya fever was suspected. Here we report a chikungunya fever outbreak in southern Mexico, involving CHIKV of Asian lineage as the etiologic agent.

## The Study

**Figure 1:**
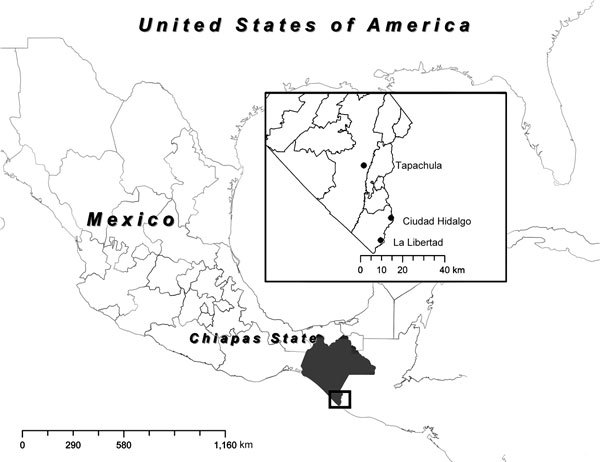
Map of Mexico showing the 3 sites where serum samples were obtained to test for chikungunya virus in Chiapas, Mexico, 2014: Tapachula, La Libertad, and Ciudad Hidalgo.

To ascertain the etiologic agent causing an outbreak of febrile illness with symptoms similar to chikungunya fever, we selected 3 sites in Chiapas State, Mexico, for sampling: Tapachula, La Libertad, and Ciudad Hidalgo (Figure 1, panel A). After patients’ informed consent was obtained, blood samples were collected from persons whose condition met the following case definition for possible chikungunya fever: acute onset of fever >38.5°C, accompanied by severe arthralgia not explained by other medical conditions (*6*). Samples from Tapachula were collected from patients who sought treatment at the Centro Regional de Investigacion en Salud Publica, whereas in La Libertad and Ciudad Hidalgo, researchers surveyed houses to identify potential case-patients. In total, 119 blood samples were collected, and serum was isolated by centrifugation. Six samples were stored in MagnaPure LC buffer (Roche, Nutley, NJ, USA), which inactivates virus particles but preserves the genomic RNA.

Viral RNA was extracted from serum samples using the ZR-96 Viral RNA Kit (Zymo Research, Orange, CA, USA) according to the manufacturer’s protocol. One-step quantitative reverse transcription PCR (qRT-PCR) (*7*) was performed by using the TaqMan RNA-to-C_t_ 1-step Kit (Applied Biosystems, San Francisco, CA, USA). Standard plaque assays were performed for the samples positive by qRT-PCR with Vero cells. Anti-CHIKV IgM-capture enzyme-linked immunosorbent assays (ELISAs) (*8*) were performed by using a chimeric Eilat-CHIKV (*9*) that contained the nonstructural proteins from Eilat virus and structural proteins from CHIKV, resulting in a structure indistinguishable from that of CHIKV. Plaque-reduction neutralization tests were used to confirm ELISA results. A sample was considered to be CHIKV negative if the sample was not positive by qRT-PCR or IgM ELISA.

Viral RNA from 5 serum samples was sent for Illumina deep sequencing and assembled by using the virus-specific HIVE-Hexagon algorithm (*10*) and the NGen module in Lasergene Suite version 10 (Bioinformatics Pioneer DNAStar, Inc., Madison, WI, USA ). Single nucleotide polymorphisms were analyzed by using the sequencing profiling tool in the HIVE suite of programs (*10*). Sequences were aligned in SeaView (*11*) by using translated proteins for the open reading frames and using nucleotides for the untranslated genome regions, and all gaps were removed. Bayesian phylogenetic inference was performed by using the general time reversible plus invariant sites plus gamma distribution 4 model in MrBayes (*12*) with 1.5 million iterations to reach congruence. Partial genome sequencing of the E2 and E1 glycoproteins was performed by using traditional Sanger methods on PCR amplicons on an additional 8 samples.

**Figure 2:**
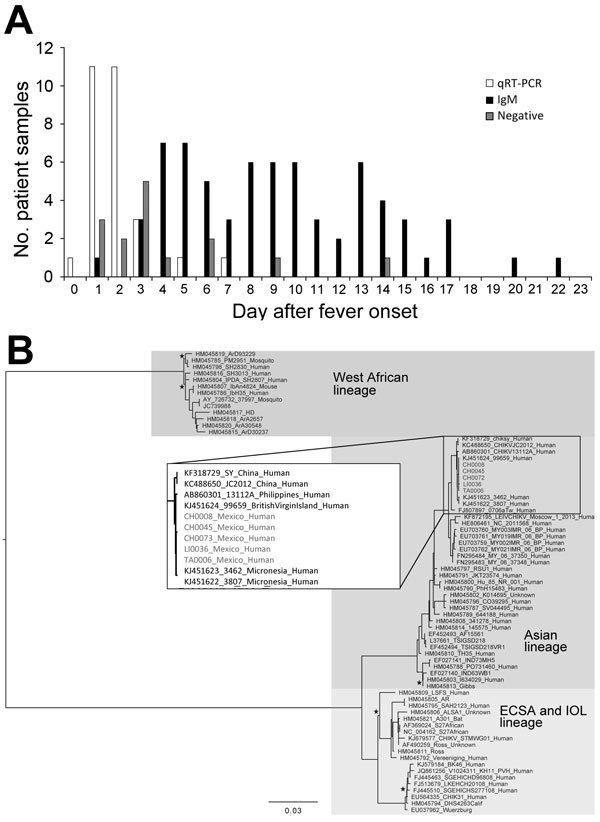
A) Number of serum samples positive for chikungunya virus (CHIKV) by reverse transcription quantitative PCR (qRT-PCR), for CHIKV IgM by ELISA, and negative for CHIKV by both methods, arranged according to day after fever onset. B) Phylogenetic tree generated by Bayesian analysis showing the relationship of the complete genomic sequences of 5 chikungunya virus isolates from Mexico and representative sequences from the GenBank library. All nodes showed posterior probabilities of >0.9, except those indicated with a star. The inset shows the closest relatives of the 5 isolates. ECSA, East/Central/South African, IOL, Indian Ocean lineage. Scale bars indicates nucleotide substitutions per site.

Over 100 serum samples were obtained from persons seeking treatment for chikungunya fever–like illness during October–December 2014 in 3 locations in Chiapas, Mexico (Tapachula, La Libertad, Ciudad Hidalgo) (Figure 1). These samples were analyzed by qRT-PCR and IgM-capture ELISA to detect viremia and IgM, respectively. No overlap occurred between the samples that were positive for CHIKV by qRT-PCR or those positive by IgM, demonstrating the importance of the humoral response to viral clearance. With few exceptions, viremia was detectable up to 3 days after fever onset (Figure 2, panel A), after which most samples were IgM positive. All age groups were equally likely to be infected, as expected during infectious disease outbreaks involving a naive population (*13*). 

Plaque assays were performed to determine serum virus titers (Table); 3 qRT-PCR samples from Tapachula could not be assayed because of sample limitations. Mean viremia level was similar among the 3 sampled locations and ranged from <2 log_10_ to 4.2 log_10_ PFU/mL.

**Table:** Proportions of CHIKV viremia and IgM in 119 serum samples collected at 3 collection sites in Chiapas, Mexico, October–December 2014*

Collection site	No. serum samples	% qRT-PCR positive serum samples (no.)	Mean serum virus titer, log_10_ PFU/mL (±SD)	% IgM-positive serum samples† (no.)
La Libertad	43	20 (9)	3.26 (0.57)	51.2 (22)
Ciudad Hidalgo	63	23.8 (15)	3.36 (0.56)	68.2 (43)
Tapachula	13	30.8 (4)	3.66	7.7 (1)

*CHIKV, chikungunya virus; qRT-PCR, quantitative reverse transcription PCR. †Six patient samples that were negative for CHIKV by qRT-PCR were unable to be used for plaque assays or ELISAs because they were stored in MagnaPure Buffer (Roche, Nutley, NJ, USA).

Five serum samples from diverse locations and collection dates were selected for Illumina sequencing (GenBank accession nos. KT327163–KT327167). The strains circulating in Chiapas were most closely related to Asian lineage strains first detected in the Caribbean (represented by a British Virgin Islands isolate) and now presumed to be circulating in much of Latin America (Figure 2, panel B). Curiously, no novel mutations appear to have been fixed in the year since the British Virgin Islands isolate was collected.

Although the genomic sequences confirmed that the circulating virus in Chiapas belonged to the Asian lineage, which is primarily transmitted by *A. aegypti* mosquitoes (*1*), we nevertheless examined the sequences for mutations known to adapt CHIKV for transmission by *A. albopictus* mosquitoes. Because both mosquito species are found in Chiapas (*14*), adaptation of the CHIKV strain circulating in Mexico to *A. albopictus* mosquitoes could place temperate regions of the eastern United States and millions of naive persons at risk for infection. None of the E2 or E1 substitutions previously reported to increase fitness in *A. albopictus* mosquitoes was observed in 8 additional samples analyzed by Sanger sequencing (GenBank accession nos. KT247378–KT247385) (*15*). One sample had a nonsynonymous mutation, in comparison to the January 2014 British Virgin Islands isolate (GenBank accession no. KJ451624) that encoded E1-V7M, which had not previously been described.

## Conclusions

We found that 79% of febrile illness cases with polyarthralgia in Chiapas State during late 2014 were caused by CHIKV. Our sequencing of CHIKV genomes confirmed spread of an Asian lineage strain from the Caribbean and suggested that although CHIKV has circulated in the Americas since 2013, no adaptive mutations have occurred. However, continued screening for vector-adaptive mutations will be critical, especially now that strains of the ECSA lineage, which gave rise to the Indian Ocean lineage, have been introduced into Brazil (*4*).
